# Redetection and description of the European dagger nematode *Xiphinema diversicaudatum* on peach (*Prunus persica* L.) in Canada

**DOI:** 10.2478/jofnem-2024-0010

**Published:** 2024-03-21

**Authors:** Jerry Akanwari, Qing Yu, Tahera Sultana

**Affiliations:** London Research and Development Center, Vineland Station, Agriculture and Agri-Food, Canada, ON, Canada; Department of Biological Sciences, Brock University, ON, Canada; Ottawa Research and Development Center, Agriculture and Agri-Food Canada, Ottawa, ON, Canada

**Keywords:** *Xiphinema diversicaudatum*, molecular characterization, phylogenetic analysis, Peach

## Abstract

The study reports the detection of *Xiphinema diversicaudatum* in a peach field in Ontario, Canada. Comprehensive population characterization involved morphological and molecular analyses using 18S and 28S rDNA sequences. Morphological and molecular analysis demonstrated a close relationship between the Ontario population and those from Central Europe. This is the first report of *X. diversicaudatum* from peaches (*Prunus persica*) in Canada and in North America.

Tender fruit production plays a significant role in Ontario’s agricultural economy, contributing about $100 million annually (OMAFRA, 2004). Despite the importance of the province to the Agricultural sector, recent observations show the presence of plant parasitic nematodes and their related damages are on the increase and could pose multifaceted challenges to the thriving peach industry (Akanwari et al., 2023). The European dagger nematode *Xiphinema diversicaudatum* (Micoletzky, 1927) Thorne, 1939, is one of the most significant species within the dagger group (*Xiphinema* spp.). The nematode parasitizes several crops, such as roses, weeds, vegetables, cover crops, potatoes, and peaches (CABI, 2021). Notably, *X. diversicaudatum* is known to vector Arabis mosaic virus in grapevines and Strawberry latent ringspot virus in strawberries (Brown, 1985; Abelleira et al., 2010). While *X. diversicaudatum* is predominantly found in Europe, it has been reported in other continents (Chizhov et al., 2014; CABI, 2021). In North America, its distribution is limited to California (USA) and Ontario (Canada) (EPPO, 2022). The nematode is listed as a regulated pest by the Canadian Food Inspection Agency (CFIA, 2023). It was first detected in Ontario in 1961, associated with roses in a commercial greenhouse (Mulvey, 1961). In 1983, it was formally declared eradicated in Canada, and since then, no documented instances of *X. diversicaudatum* have been reported in the country (Brown, 1983; CABI, 2021).

During a recent nematode study on peaches in Ontario, we detected the presence of a *Xiphinema* species resembling *X. diversicaudatum*. Therefore, the main objectives of this study were to provide a detailed description of the *Xiphinema* species using an integrative approach of morphological and molecular characterization of the 18s DNA rRNA. Soil samples were collected from the rhizosphere of peach (*Prunus persica*) plants in Southern Ontario, Canada at 30 cm depth. Nematodes extracted (100 g) using the sucrose centrifugal flotation (Jenkins, 1964) and then fixed in TAF (triethanolamine formalin). The fixed nematodes were processed to glycerine and mounted on slides for detailed compound microscopic studies (Eisenback and Hunt, 2021). The female had the following measurement (Figures 1A–D): body length 4829 ± 91μm (4700–4900 μm), a (77.1 ± 1.2 μm, 75.2–78 μm), b (10.3 ± 0.1 μm,10.3–10 μm), c (99.3 ± 1.7 μm, 98.3–102 μm), total stylet length (220 ± 3.5 μm, 217–225 μm), and tail (48 ± 1.7 μm, 46–50 μm). The dorsal and sub-ventral glands are situated near the anterior end of the esophagus base, which is 113–114 µm long and 23–24 µm wide. The gonads are symmetric and didelphic, with reflexed ovaries and spindle-shaped, sperm-filled genital tracts. The males had a slightly shorter body (4463 ± 97 µm, 4390–4600 µm) and shorter odontostyles (123–130 µm). Tails are like female tails with pegs, but the ventral sides are more flattened, and in some specimens, the pegs are very short (Figures 1E,F). The tail of the third stage juvenile was not pegged as described (Micoletzky, 1927) Thorne, 1939, but was in agreement with the redescription by Goodey et al. (1960) and Barsi and Lamberti (2000), based on a different population.

**Figure 1:**
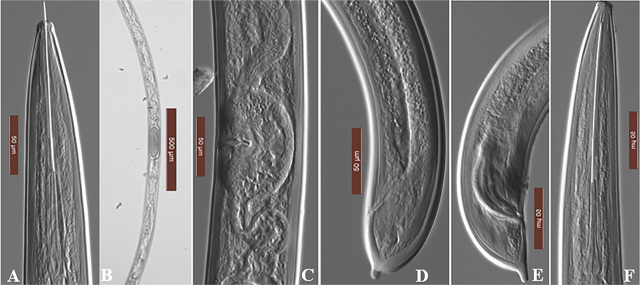
Micrographs of *Xiphinema diversicaudatum*: A–C: female anterior region; D: female posterior region; E: male posterior region, F: male anterior region.

DNA extraction was done on individual juveniles using primer sets 18S-1813F/18S-2646R (Holterman et al., 2006) and 28S-CL-F2/28S-CL-R1 (Carta and Li, 2019). The polymerase chain reaction (PCR) conditions and direct DNA sequencing was performed following Carta and Li, 2019. The DNA sequences were deposited at GenBank with accession numbers OR192925, OR195517, and OR195518. The top 18S and 28S expansion segment BLASTN hit was *X. diversicaudatum* from the Czech Republic with percentage identities ranging from 99.46–100% at 100% query coverage.

The molecular analysis of the 18S and 28S rDNA closely resembled *X. diversicaudatum* from the Czech Republic. The identification of *X. diversicaudatum* in Ontario marks its reappearance in Canada. Therefore, a comprehensive assessment of the economic impact of *X. diversicaudatum* and its virus transmission on Canadian agriculture is needed. To the best of our knowledge, this is the first record of *X. diversicaudatum* parasitizing the roots of peaches in Canada and North America.
